# Molecular Pathogenesis of Memory Impairment in Parkinson's Disease: An Exploration of Underlying Mechanisms

**DOI:** 10.1002/hsr2.72024

**Published:** 2026-04-21

**Authors:** Mohammad Pourfridoni, Zohreh Hakemi, Habibeh Mashayekhi‐sardoo, Yousef Baghcheghi

**Affiliations:** ^1^ Department of Otolaryngology, Head and Neck Surgery, Otorhinolaryngology Research Center Shahid Sadoughi University of Medical Sciences Yazd Iran; ^2^ Student Research Committee Jiroft University of Medical Sciences Jiroft Iran; ^3^ Bio Environmental Health Hazards Research Center Jiroft University of Medical Sciences Jiroft Iran

**Keywords:** memory disorders, molecular biology, Parkinson's disease, pathogenesis

## Abstract

**Background and Aim:**

Parkinson's disease (PD) is primarily known for its motor symptoms but is increasingly recognized for its substantial cognitive effects, particularly on memory. This narrative review aims to clarify the complex molecular mechanisms contributing to memory impairment in PD by integrating findings from both animal and clinical research.

**Methods:**

This study is a narrative review synthesizing data from existing literature. It begins by addressing the prevalence of cognitive deficits among PD patients. It then explores animal studies investigating pathophysiological changes associated with PD, focusing on the impact of dopaminergic depletion on neural circuitry. These findings are integrated with clinical research to identify specific molecular changes closely associated with memory impairment independent of motor dysfunction.

**Results:**

The review systematically examines key molecular mechanisms contributing to memory deficits in PD. These mechanisms include disruptions in neural circuits, dopamine depletion, neuroinflammation, oxidative stress, and alpha‐synuclein pathology. Additional contributing factors identified include cholinergic system dysfunction, cortical thinning and atrophy, disrupted neural oscillations, gut‐brain axis dysfunction, impaired synaptic plasticity, and alterations in neurotransmitter systems. The analysis illustrates how these factors collectively undermine cognitive function.

**Conclusion:**

By synthesizing insights from various studies, this narrative review offers a comprehensive perspective on the intricate molecular pathogenesis of memory impairment in PD. It underscores the necessity of addressing both motor and non‐motor symptoms in managing PD effectively and advocates for a holistic treatment approach that considers cognitive decline alongside traditional motor symptom management.

**Abbreviations**
5‐HT: 5‐Hydroxytryptamine
6‐OHDA: 6‐hydroxydopamine
Ach: Acetylcholine
AMPA: Alpha‐amino‐3‐hydroxy‐5‐methyl‐4‐isoxazole propionate
BDNF: Brain‐derived neurotrophic factor
CaMKII: Calcium/calmodulin‐dependent protein kinase II
CAT: Catalase
CNS: Central Nervous System
COX2: Cyclooxygenase 2
DAMP: Damage‐associated molecular pattern
DAT: Dopamine transporter
DLB: Dementia with Lewy bodies
GABA: Gamma‐Aminobutyric Acid
GBA: Glucosylceramidase beta
GFAP: Glial Fibrillary Acidic Protein
HPA: Hypothalamic‐pituitary‐adrenal
IEGs: Immediate early genes
IL‐1: Interleukin‐1
IL‐6: Interleukin‐6.
LRRK2: Leucine Rich Repeat Kinase 2
LTD: Long‐term depression
LTP: Long‐term potentiation
MDA: Malondialdehyde
MPP+: 1‐methyl‐4‐phenylpyridinium
MPTP: 1‐methyl‐4‐phenyl‐1,2,3,6‐tetrahydropyridine
MRI: Magnetic resonance imaging
NE: Norepinephrine
NF‐kB: Nuclear factor kB
NMDA: N‐methyl‐d‐aspartate
NO: Nitric oxide,
NSAIDs: Nonsteroidal anti‐inflammatory drugs
PARK7: Parkinsonism Associated Deglycase
PD: Parkinson Disease
PDD: PD with dementia
PET: Positron emission tomography
ROS: Reactive oxygen species
SCFAs: Short‐chain fatty acids
SERT: Serotonin transporter
SNCA: Synuclein Alpha
SOD: Superoxide dismutase
tACS: Transcranial alternating current stimulation
TLR: Toll‐like receptor, ↓: Decrease; ↑: Increase
TLRs: Toll‐like receptors
TMS: Transcranial magnetic stimulation
TNF‐α: Tumor necrosis factor‐alpha

## 1 Introduction

Parkinson's disease (PD) is a progressive neurodegenerative disorder characterized by motor symptoms such as bradykinesia, tremor, rigidity, and postural instability. However, its impact extends beyond these motor manifestations, significantly affecting cognitive functions and quality of life. Memory deficits are particularly pronounced in PD patients, disrupting daily activities and overall well‐being. As the disease progresses, cognitive decline affects various domains, including executive function and attention, necessitating a deeper understanding of the molecular mechanisms behind these impairments to develop effective interventions [1, 2, 3, 4, 5].

The relationship between PD and memory impairment is complex. The degeneration of dopaminergic neurons in the substantia nigra leads to cascading effects on brain regions involved in cognitive processing. Disruption in neurotransmitter systems, particularly dopamine and acetylcholine, plays a crucial role in cognitive decline. Dopamine depletion affects motivation and learning, while compromised cholinergic systems hinder attention and memory consolidation. This neurotransmitter dysregulation creates an environment that impairs optimal cognitive functioning [6, 7].

Additionally, the aggregation of α‐synuclein protein in Lewy bodies contributes to cognitive decline by disrupting synaptic function and promoting neuroinflammation. Neuroinflammation, characterized by microglial activation and pro‐inflammatory cytokine release, exacerbates neuronal loss and synaptic disconnection. The interplay of genetic predispositions, environmental factors, and lifestyle choices also complicates our understanding of memory impairment in PD. Factors such as physical activity and dietary habits may influence cognitive health, presenting potential avenues for intervention [8, 9, 10, 11, 12].

Understanding the molecular mechanisms underlying memory impairment in PD is vital for developing targeted therapies and identifying biomarkers for early detection. While the primary focus of this review is on PD, it is noteworthy that several core mechanisms discussed herein—such as neuroinflammation, oxidative stress, and impaired neurotrophic signaling—represent common pathological themes also observed in memory dysfunction induced by diverse etiologies, including chronic ketamine exposure, obesity, and toxic cannabinoid use [13, 14, 15]. This review aims to explore the connections between PD and memory impairment by examining pathways involved in dopaminergic neuron degeneration, α‐synuclein aggregation, neurotransmitter dysregulation, and neuroinflammatory processes. By synthesizing existing literature on these mechanisms and considering genetic and environmental influences, we hope to enhance understanding of cognitive challenges faced by individuals with PD and contribute to better management strategies for improving patient outcomes.

## 2 Animal Studies

Animal studies have played a pivotal role in advancing our understanding of the molecular mechanisms behind memory impairment associated with PD. These studies utilize various animal models, particularly rodents, to replicate key features of PD, including both motor deficits and cognitive impairments. One prominent model is the 6‐hydroxydopamine (6‐OHDA) lesion model, which selectively targets dopaminergic neurons in the substantia nigra, leading to significant dopamine depletion. This model not only mimics the hallmark motor symptoms of PD but also exhibits cognitive deficits similar to those observed in human patients. Research has demonstrated that the loss of dopaminergic neurons correlates with alterations in neurotransmitter systems, particularly glutamate and acetylcholine, which are vital for memory and learning processes [16, 17, 18, 19, 20, 21].

In addition to the 6‐OHDA model, the 1‐methyl‐4‐phenyl‐1,2,3,6‐tetrahydropyridine (MPTP) model has been instrumental in elucidating the molecular underpinnings of memory impairment in PD. MPTP induces parkinsonian symptoms by selectively causing degeneration of dopaminergic neurons. Studies using this model have shown that the neuroinflammatory response triggered by dopaminergic cell death significantly contributes to cognitive deficits. Elevated levels of pro‐inflammatory cytokines and activated microglia have been observed in MPTP‐treated animals, indicating that neuroinflammation may play a critical role in the progression of memory impairment in PD [22, 23, 24].

Genetic models of PD, particularly those involving mutations in the alpha‐synuclein gene, have also provided insights into memory impairment mechanisms. These models exhibit accumulation of alpha‐synuclein aggregates characteristic of PD pathology, correlating with synaptic dysfunction and cognitive decline. Disruption of synaptic integrity and impairment of long‐term potentiation have been documented in these models, highlighting the importance of alpha‐synuclein in maintaining cognitive function [25, 26, 27]. Furthermore, therapeutic interventions tested in animal studies have shown promise; pharmacological agents like levodopa demonstrate varying effects on cognitive function, while non‐pharmacological strategies such as exercise and cognitive training have been linked to improved cognitive outcomes through enhanced neurogenesis and synaptic plasticity [28, 29, 30, 31].

In conclusion, animal studies have significantly enriched our understanding of the connections between PD and memory impairment. By utilizing various models, researchers have elucidated the roles of neurotransmitter systems, neuroinflammation, and genetic factors in cognitive decline. The insights gained from these studies pave the way for developing targeted therapeutic strategies aimed at preserving cognitive function in individuals with PD. As research continues to evolve, integrating findings from animal studies with clinical observations will be essential for unraveling the complexities surrounding memory impairment in PD and improving patient outcomes.

## 3 Clinical Studies

Clinical studies have significantly enhanced our understanding of PD, a neurodegenerative disorder primarily characterized by motor symptoms such as tremors, rigidity, and bradykinesia. Increasingly, attention is being drawn to its non‐motor symptoms, particularly cognitive impairments and memory deficits. Research indicates that cognitive decline is common in PD, affecting memory, executive function, and attention. A longitudinal study by Aarsland et al. (2010) revealed that approximately 30% to 40% of patients develop dementia within 10 years of diagnosis, highlighting the urgent need to understand the underlying mechanisms contributing to cognitive decline [32]. Neuroimaging studies utilizing positron emission tomography (PET) and magnetic resonance imaging (MRI) have identified alterations in brain regions associated with memory, particularly the hippocampus and prefrontal cortex [33].

Clinical trials assessing dopaminergic therapies like levodopa have produced mixed results regarding their impact on cognitive function. While levodopa effectively alleviates motor symptoms, its effects on cognitive decline remain debated. For instance, a study by Molloy et al. indicated that after 3 months of treatment, patients experienced declines in verbal attention and memory despite improvements in overall cognitive function. In patients with Parkinson's Disease dementia (PDD), slower reaction times were noted in specific cognitive tests, though significant cognitive deterioration was not observed. In contrast, patients with dementia with Lewy bodies (DLB) did not exhibit adverse cognitive effects from levodopa treatment. These findings underscore the complexity of levodopa's effects on cognition and suggest the need for exploring alternative therapeutic options like cholinesterase inhibitors and glutamatergic agents to enhance cognitive outcomes in PD patients [34, 35].

Non‐pharmacological approaches such as cognitive training and physical exercise have also been investigated for their potential to mitigate memory impairment in PD. A randomized controlled trial by Li et al. (2016) demonstrated that structured cognitive training significantly improved memory performance in PD patients compared to a control group. Additionally, regular physical activity has been shown to enhance cognitive function and promote neuroplasticity, potentially counteracting some of the cognitive decline associated with PD [36]. Neuroinflammation has emerged as a critical factor contributing to cognitive decline in PD; elevated levels of pro‐inflammatory cytokines in the cerebrospinal fluid correlate with cognitive impairment. Research by Tansey et al. (2013) emphasized microglial activation's role in neurodegeneration and cognitive decline progression, suggesting that targeting neuroinflammatory pathways may offer new therapeutic avenues for addressing memory impairment in PD [37].

Genetic studies have identified risk factors associated with cognitive decline in PD, including mutations in genes such as glucosylceramidase beta (GBA) and LRRK2. These genetic factors may influence neurodegeneration and neuroinflammation pathways, complicating the relationship between PD and memory impairment. Understanding these genetic links is essential for developing personalized treatment strategies tailored to individual needs [38, 39, 40]. In summary, clinical studies have greatly advanced our understanding of the molecular mechanisms linking PD to memory impairment. The interplay among neurodegeneration, neurotransmitter dysregulation, neuroinflammation, and genetic factors highlights the complexity of cognitive decline in PD. Ongoing research into innovative therapeutic strategies that address both motor symptoms and cognitive challenges will be vital for improving quality of life for patients and their families while enhancing management of this multifaceted disorder.

## 4 Molecular Mechanisms

### 4.1 Disruption of Neural Circuits

PD is a neurodegenerative disorder primarily affecting dopaminergic neurons in the substantia nigra, leading to a cascade of molecular and cellular changes that disrupt neural connectivity and function. These disruptions extend beyond motor symptoms, significantly impacting memory formation, retrieval, and overall cognitive function. At the core of PD‐related cognitive decline is the loss of dopaminergic neurons, which diminishes dopamine levels in critical brain areas like the striatum and prefrontal cortex. Dopamine is essential for modulating synaptic activity and facilitating communication within neural circuits, and its depletion disrupts the balance of excitatory and inhibitory signals, impairing neural networks involved in learning and memory [41, 42, 43, 44].

A significant molecular player in the disruption of neural circuits in PD is α‐synuclein. This protein aggregates in Lewy bodies, interfering with synaptic vesicle release mechanisms and altering neurotransmitter dynamics. Aggregated α‐synuclein inhibits the release of dopamine and glutamate, disrupting long‐term potentiation (LTP), a key process for synaptic strengthening that underlies memory formation. Additionally, α‐synuclein aggregates contribute to neuroinflammation, exacerbating neural circuit disruption through chronic inflammation and microglial activation, leading to further neuronal damage and loss [45, 46, 47, 48].

Dysregulation of glutamatergic signaling also contributes to disrupted neural circuits in PD. Glutamate is the primary excitatory neurotransmitter, essential for normal cognitive function. In PD, an imbalance in glutamate levels can lead to excitotoxicity, damaging neurons and impairing synaptic plasticity by affecting NMDA receptors. This excitotoxicity can induce long‐term depression (LTD), further contributing to memory impairments [46]. Moreover, the cholinergic system plays a crucial role in cognitive functions like attention, learning, and memory. Loss of cholinergic signaling in PD can lead to deficits in these processes, and the interplay between dopaminergic and cholinergic systems is vital for maintaining circuit integrity [49, 50].

The cumulative effects of dopamine depletion, α‐synuclein aggregation, neuroinflammation, and neurotransmitter dysregulation create a complex environment that perpetuates cognitive decline in PD. Understanding these molecular mechanisms is essential for developing targeted therapies to mitigate memory impairments and improve cognitive outcomes in individuals with PD. Clinical and animal studies have provided valuable insights into these mechanisms, highlighting the need for comprehensive therapeutic strategies that address both motor and cognitive symptoms of PD. Disruption of neural circuits is a critical mechanism underlying cognitive decline in PD. The loss of dopaminergic neurons in the substantia nigra leads to decreased dopamine levels, particularly affecting brain regions such as the hippocampus and prefrontal cortex, which are integral to memory formation and retrieval [51].

This neurotransmitter imbalance disrupts synaptic activity, impairing communication within neural circuits essential for learning and memory. The interplay between these neurotransmitter systems, especially the dopaminergic and cholinergic systems, is vital for maintaining cognitive function. Research indicates that alterations in these systems can lead to significant cognitive deficits in PD patients.

Neurotrophic factors like brain‐derived neurotrophic factor (BDNF) also play a crucial role in maintaining neural circuit integrity. BDNF is essential for neuron survival, growth, and synaptic plasticity; however, its levels are often reduced in PD due to neuroinflammatory processes and neuronal loss [52]. This downregulation impairs mechanisms necessary for synaptic strengthening and maintenance, further disrupting the circuits required for memory function. Additionally, low BDNF levels increase neuronal vulnerability to degeneration, perpetuating cognitive decline.

The effects of disrupted neural circuits extend to alterations in functional connectivity between brain regions involved in memory processing. Neuroimaging studies have shown that PD patients often exhibit altered connectivity patterns between the hippocampus, prefrontal cortex, and other critical areas for memory functions. Reduced functional connectivity has been associated with impaired memory performance among these patients. Such changes can stem from both structural atrophy of specific brain regions and functional alterations in neural activity, leading to inefficient information processing and retrieval [53, 54].

Moreover, lifestyle factors such as physical activity play a significant role in modulating cognitive function in PD. Regular exercise has been shown to enhance neuroplasticity and improve cognitive outcomes, while sedentary behavior can exacerbate cognitive declines. Dietary factors may also influence neuroinflammation and neurotrophic factor availability, further impacting cognitive health [15, 29, 30]. In conclusion, understanding the complex interplay of neurotransmitter imbalances, neurotrophic factor signaling, and lifestyle influences is essential for developing therapeutic strategies aimed at mitigating cognitive decline in individuals with PD.

### 4.2 Dopamine Depletion

Dopamine depletion is a critical mechanism in PD, significantly contributing to both cognitive and motor deficits associated with the disorder. The loss of dopaminergic neurons, particularly in the substantia nigra pars compacta, leads to a substantial decrease in dopamine levels across the striatum and other brain regions. This depletion profoundly affects neural circuitry, especially in areas involved in memory and executive functions. Understanding the molecular mechanisms underlying dopamine depletion in PD is essential for elucidating the cognitive impairments that accompany this neurodegenerative disorder.

Dopamine is synthesized from the precursor amino acid tyrosine through enzymatic processes involving tyrosine hydroxylase and aromatic l‐amino acid decarboxylase. In a healthy brain, precise regulation of dopamine synthesis, release, and reuptake is crucial for maintaining optimal levels of this neurotransmitter. However, in PD, various pathological processes contribute to dopamine depletion. Key factors include the degeneration of dopaminergic neurons due to genetic predispositions, environmental toxins, and age‐related factors, which reduce the number of neurons capable of producing dopamine [55].

The aggregation of α‐synuclein, a protein that forms Lewy bodies, significantly contributes to dopamine depletion. The misfolding and accumulation of α‐synuclein disrupt normal cellular processes within dopaminergic neurons, impairing mitochondrial function and increasing oxidative stress. Mitochondrial dysfunction can initiate a cascade of events leading to neuronal damage and apoptosis. Additionally, α‐synuclein aggregates disrupt proteasomal and autophagic pathways responsible for clearing damaged proteins, leading to toxic environments within neurons that exacerbate dopamine loss [56, 57]. Neuroinflammation also plays a critical role; activated microglia release pro‐inflammatory cytokines and reactive oxygen species that further impair dopaminergic neurons and accelerate dopamine depletion [11, 12].

Moreover, dysregulation of neurotransmitter systems such as glutamate and acetylcholine impacts dopamine levels. In PD, excessive glutamatergic activity can lead to excitotoxicity, causing neuronal damage through overstimulation of NMDA receptors. This excitotoxic environment impairs remaining dopaminergic neurons and contributes to further dopamine depletion [46]. The loss of acetylcholine signaling from the cholinergic system also influences dopamine dynamics; its depletion can alter dopamine release and uptake, highlighting the complex interplay between these neurotransmitter systems [58]. Understanding these mechanisms is vital for developing therapeutic strategies aimed at mitigating cognitive decline in individuals affected by PD.

In the hippocampus, dopamine is essential for encoding and consolidating new memories, as dopaminergic input is necessary for long‐term potentiation (LTP), the process that strengthens synaptic connections critical for memory formation [59]. Decreased dopamine levels compromise mechanisms supporting LTP, leading to difficulties in forming new memories and retrieving existing ones. This impairment is often linked to altered glutamatergic signaling, as dopamine modulates the release of glutamate, the brain's primary excitatory neurotransmitter. A decline in dopamine can reduce glutamatergic activity, further hindering synaptic plasticity and memory processes [60].

The consequences of dopamine depletion extend significantly to cognitive processes such as memory. Dopamine is pivotal in motivation, reinforcement learning, and the encoding of new memories [61]. The striatum, a key region affected by dopamine depletion, processes reward‐related information and integrates it into memory formation. When dopamine levels are low, encoding and retrieving memories becomes impaired, resulting in challenges in learning new information and recalling previously learned material [62]. Additionally, the prefrontal cortex, heavily influenced by dopaminergic signaling, is critical for higher‐order cognitive functions such as executive function and working memory. Dopamine depletion in this area can lead to deficits in attention, planning, and decision‐making, compounding cognitive impairments experienced by individuals with PD.

The interplay between dopamine depletion and other factors contributing to cognitive decline in PD is complex. Non‐motor symptoms such as depression and anxiety can exacerbate cognitive deficits. These mood disorders are often linked to changes in dopaminergic signaling and create additional challenges for individuals coping with PD [63, 64]. The interaction among mood, motivation, and cognitive function underscores the necessity of addressing these issues holistically during treatment.

In summary, dopamine depletion is a central feature of PD driven by neurodegeneration, α‐synuclein aggregation, mitochondrial dysfunction, neuroinflammation, and neurotransmitter dysregulation. The resulting loss of dopamine profoundly impacts cognitive processes—particularly memory—by disrupting neural circuits underlying these functions. Understanding the molecular mechanisms contributing to dopamine depletion is crucial for developing targeted therapies aimed at mitigating cognitive decline in individuals with PD. By addressing the complexities of dopamine dynamics and their role in cognitive function, researchers and clinicians can work toward improved strategies for managing the cognitive challenges faced by those affected by this debilitating disorder.

### 4.3 Oxidative Stress

Oxidative stress is a significant pathological process in PD, contributing to neuronal degeneration and cognitive impairments. It arises from an imbalance between the production of reactive oxygen species (ROS) and the body's ability to detoxify these reactive intermediates or repair the resulting damage. In PD, oxidative stress plays a critical role in the neurodegenerative processes affecting dopaminergic neurons and other brain regions involved in memory and cognitive function. Understanding the molecular mechanisms leading to oxidative stress in PD is essential for uncovering its implications for neuronal health and cognitive outcomes.

The generation of ROS, including free radicals such as superoxide anions and hydroxyl radicals, primarily occurs as a byproduct of normal cellular metabolism, particularly in mitochondria during oxidative phosphorylation. In healthy neurons, a delicate balance is maintained between ROS production and antioxidant defenses that neutralize these reactive species [65, 66]. However, in PD, several factors contribute to increased ROS production and depletion of antioxidant defenses, culminating in oxidative stress [67, 68]. Mitochondrial dysfunction is a primary contributor; the degeneration of dopaminergic neurons is often associated with mitochondrial impairment due to the accumulation of misfolded proteins like α‐synuclein. This aggregation disrupts mitochondrial dynamics and function, leading to impaired ATP production and increased ROS release [69].

Additionally, exposure to neurotoxic substances such as 1‐methyl‐4‐phenylpyridinium (MPP + ) exacerbates oxidative stress by selectively targeting dopaminergic neurons and inhibiting mitochondrial function. This inhibition diminishes ATP production while increasing ROS generation, initiating lipid peroxidation, protein oxidation, and DNA damage—collectively compromising neuronal integrity [70, 71]. The inflammatory response in the brain further contributes to oxidative stress; microglial activation in response to neuronal injury can lead to the release of pro‐inflammatory cytokines and additional ROS, perpetuating oxidative damage and creating a toxic environment for surviving neurons [45, 72].

Antioxidant defenses are crucial for mitigating oxidative stress. In healthy neurons, endogenous antioxidants such as glutathione, superoxide dismutase (SOD), and catalase neutralize ROS. However, in PD, levels of these antioxidants are often reduced or impaired, leading to decreased capacity to combat oxidative stress. For instance, glutathione is frequently found depleted in the brains of PD patients due to its consumption outpacing replenishment [73]. The molecular consequences of oxidative stress are profound; it can lead to oxidation of lipids, proteins, and nucleic acids, resulting in cellular dysfunction. Lipid peroxidation compromises cellular membranes, impairing synaptic function essential for cognitive processing. Furthermore, oxidative stress can induce mitochondrial apoptosis—a programmed form of cell death—leading to significant neuronal loss in regions critical for memory and cognition.

Emerging evidence suggests that oxidative stress significantly influences neurogenesis and synaptic plasticity, which are critical for learning and memory [74]. In the context of PD, oxidative stress can impair these processes, hindering the brain's ability to adapt and respond to new information. This impairment may exacerbate memory deficits in individuals with PD, highlighting the importance of understanding how oxidative stress affects cognitive function.

Lifestyle factors have been increasingly recognized for their role in modulating oxidative stress in PD. Regular physical activity and antioxidant‐rich diets have been associated with reduced oxidative stress and improved cognitive outcomes. Exercise enhances mitochondrial function and promotes the expression of endogenous antioxidants, thereby mitigating the effects of oxidative stress [75, 76]. Dietary interventions, such as adopting a Mediterranean diet rich in fruits, vegetables, and healthy fats, may provide neuroprotective benefits through their antioxidant properties [77].

### 4.4 Neuroinflammation

Neuroinflammation has emerged as a critical component in the pathophysiology of PD, significantly contributing to the cognitive impairments and neurodegeneration associated with the disorder. This inflammatory response within the central nervous system (CNS) is characterized by the activation of glial cells, particularly microglia and astrocytes, which are essential for maintaining homeostasis and responding to injury. However, in PD, neuroinflammation becomes dysregulated, leading to a chronic inflammatory state that exacerbates neuronal damage and cognitive decline. Understanding the molecular mechanisms underlying neuroinflammation in PD is essential for elucidating how this process contributes to memory impairment and identifying potential therapeutic targets.

At the heart of neuroinflammation in PD is the activation of microglia, the resident immune cells of the CNS. In a healthy brain, microglia continuously survey their environment, ready to respond to signs of injury or cellular distress. In PD, microglial activation is triggered by factors such as misfolded proteins like α‐synuclein, oxidative stress, and neuronal damage [11, 12]. The accumulation of α‐synuclein—a hallmark of PD—activates microglia through receptors like Toll‐like receptors (TLRs), leading to the release of pro‐inflammatory cytokines such as TNF‐α and IL‐1β. These cytokines amplify the inflammatory response and can induce apoptosis in dopaminergic neurons, impairing synaptic plasticity and contributing to memory deficits [78, 79].

Astrocytes also become activated in response to neuroinflammation in PD. While they typically support neuronal health, their activation can lead to the release of both protective and toxic factors. Activated astrocytes produce glial fibrillary acidic protein (GFAP) and secrete cytokines like IL‐1β and IL‐6, further contributing to inflammation. Dysregulated astrocytic functions can impair neurotransmitter uptake and metabolic support, hindering synaptic function and exacerbating cognitive impairments observed in PD [80, 81]. The chronic nature of neuroinflammation creates a feedback loop where pro‐inflammatory mediators lead to further neuronal injury, promoting additional microglial activation and creating an environment detrimental to neuronal survival.

Current research highlights various factors contributing to neuroinflammation in PD. Environmental toxins and genetic predispositions may influence individual susceptibility to these processes [2]. Additionally, alterations in gut microbiota have been implicated in promoting systemic inflammation that affects the CNS [5]. These findings suggest that complex interactions between genetic factors and environmental exposures may create an inflammatory environment conducive to PD progression.

One of the key molecular pathways involved in neuroinflammation associated with PD is the nuclear factor‐kappa B (NF‐κB) signaling pathway. NF‐κB acts as a transcription factor activated by pro‐inflammatory signals, regulating the expression of inflammatory genes, including cytokines and chemokines. In PD, the activation of NF‐κB in microglia and astrocytes leads to sustained production of inflammatory mediators, contributing to neuroinflammation and advancing neurodegeneration and cognitive decline [82]. Additionally, oxidative stress plays a significant role by activating microglia and astrocytes, which release pro‐inflammatory cytokines that can further exacerbate oxidative stress, creating a harmful feedback loop that accelerates neuronal damage [83].

Neuroinflammation disrupts crucial neuronal signaling pathways necessary for memory and cognitive function. Chronic release of inflammatory cytokines impairs glutamate receptor function, essential for synaptic plasticity and memory formation. This impairment can lead to deficits in long‐term potentiation (LTP), which is vital for learning and memory [84]. The hippocampus, critical for memory processing, is particularly vulnerable; studies have shown that elevated pro‐inflammatory cytokine levels correlate with hippocampal atrophy and cognitive decline in PD patients [85]. The balance between excitatory and inhibitory neurotransmission in this region is disrupted by neuroinflammatory processes, resulting in difficulties with memory consolidation and retrieval.

The effects of neuroinflammation extend beyond the hippocampus to other cognitive processing areas like the prefrontal cortex, which is crucial for executive function and decision‐making. Neuroinflammation in this region can lead to cognitive deficits that compound challenges faced by individuals with PD. Dysfunction in both the hippocampus and prefrontal cortex results in a wide range of memory impairments affecting both short‐term and long‐term capabilities [85].

Given the detrimental effects of neuroinflammation in PD, targeting this process offers a promising therapeutic avenue. Approaches aimed at modulating microglia and astrocyte activity could restore homeostasis and protect against neuronal damage. Various strategies, including nonsteroidal anti‐inflammatory drugs (NSAIDs) and cytokine inhibitors, have been explored in preclinical and clinical settings [86, 87]. Moreover, lifestyle factors such as regular physical activity and antioxidant‐rich dietary interventions may also help mitigate neuroinflammation effects, supporting cognitive health [15, 29, 88, 89]. Understanding these complex interactions is essential for developing targeted therapies that improve outcomes for those living with PD.

### 4.5 Alpha‐Synuclein Pathology

Alpha‐synuclein pathology is a central feature of PD and is closely linked to the neurodegenerative processes that contribute to both motor and cognitive impairments. This presynaptic protein is crucial for synaptic function and neurotransmitter release, but in PD, it undergoes pathological changes characterized by misfolding and aggregation into Lewy bodies. These alterations significantly affect neuronal health and memory function, making it essential to understand the molecular mechanisms associated with alpha‐synuclein pathology to address cognitive deficits in PD [90].

In healthy neurons, alpha‐synuclein exists as a soluble monomer that aids in synaptic integrity. The pathological process begins with its misfolding into oligomers and fibrils, influenced by genetic mutations, environmental toxins, and cellular stressors. Notably, mutations in the SNCA gene have been linked to familial forms of PD, enhancing the protein's tendency to aggregate [91]. The aggregation process is detrimental; oligomers are particularly neurotoxic, disrupting cellular functions and leading to oxidative stress and mitochondrial dysfunction, which further compromise neuronal integrity and impair processes essential for learning and memory [69, 71, 92].

The accumulation of alpha‐synuclein aggregates leads to the formation of Lewy bodies, which disrupt normal cellular organization and function. This is especially evident in dopaminergic neurons within the substantia nigra, where their loss contributes to motor symptoms as well as cognitive decline affecting areas like the prefrontal cortex and hippocampus [93]. Furthermore, misfolded alpha‐synuclein can activate microglia, resulting in chronic neuroinflammation that exacerbates neuronal damage and perpetuates neurodegeneration [94]. Mitochondrial dysfunction is also a critical aspect of this pathology, as aggregated alpha‐synuclein can impair mitochondrial function, leading to energy deficits and increased oxidative stress.

The spread of alpha‐synuclein pathology throughout the brain occurs in a prion‐like manner, where misfolded proteins induce neighboring neurons to misfold as well [95, 96, 97]. This intercellular transmission contributes to the progressive nature of PD. The implications for cognitive function are significant; both the prefrontal cortex and hippocampus are affected by alpha‐synuclein aggregates, leading to deficits in decision‐making, attention, learning, and memory retrieval. Understanding these complex interactions is vital for developing targeted therapies aimed at mitigating alpha‐synuclein pathology and improving cognitive outcomes for individuals with PD.

The interplay between alpha‐synuclein pathology and neuroinflammation significantly complicates our understanding of cognitive decline in PD. These two processes can create a synergistic effect, where the presence of aggregated alpha‐synuclein exacerbates neuroinflammation, leading to increased neuronal damage. This complex relationship underscores the necessity for a multifaceted approach to address cognitive impairment in PD [11, 52, 80, 85, 98]. As researchers continue to explore these interactions, it becomes clear that targeting both alpha‐synuclein and neuroinflammation may be essential for effective therapeutic strategies.

Therapeutic strategies aimed at alpha‐synuclein pathology are currently a major focus of research. Approaches include reducing alpha‐synuclein aggregation, enhancing its clearance from cells, and preventing its toxic effects. For example, small molecules that stabilize the monomeric form of alpha‐synuclein or enhance its degradation through autophagy and proteasomal pathways are being investigated as potential treatments. Additionally, immunotherapy targeting alpha‐synuclein aggregates aims to enhance the clearance of these toxic species and prevent their spread throughout the brain [99, 100]. Such strategies could mitigate the neurotoxic effects associated with aggregated forms of the protein.

Collectively, evidence confirms that alpha‐synuclein pathology is a central feature of PD that contributes significantly to neurodegenerative processes leading to cognitive impairments. The misfolding and aggregation of alpha‐synuclein disrupt synaptic function, induce neuroinflammation, impair mitochondrial health, and overwhelm cellular proteostasis mechanisms. These disruptions have profound implications for memory and cognitive processes, particularly in key brain regions essential for these functions. Understanding the molecular mechanisms underlying alpha‐synuclein pathology is critical for developing targeted therapeutic strategies aimed at mitigating its effects and improving cognitive outcomes in individuals with PD.

### 4.6 Cholinergic System Dysfunction

PD significantly disrupts cholinergic system function, which is critical for cognitive processes such as learning and memory. The cholinergic system primarily utilizes acetylcholine (ACh), synthesized by cholinergic neurons from the basal forebrain that project to areas like the hippocampus and cortex. In PD, a hallmark is the degeneration of these cholinergic neurons, particularly in the basal forebrain, leading to decreased levels of ACh. This reduction impairs cholinergic signaling, affecting synaptic plasticity, which is essential for learning and memory consolidation [58].

The molecular pathways involved in cholinergic dysfunction are intricate and interconnected. Reduced ACh levels lead to diminished activation of nicotinic and muscarinic receptors, crucial for cholinergic neurotransmission. This impairment results in weakened synaptic transmission and long‐term potentiation (LTP), further hindering the brain's capacity to form and retain memories [101]. Additionally, the interaction between cholinergic dysfunction and alpha‐synuclein pathology exacerbates cognitive decline. Alpha‐synuclein aggregates can increase oxidative stress and neuroinflammation, creating an inflammatory environment that further disrupts cholinergic circuits and contributes to memory deficits [12, 100].

As PD progresses, cognitive deficits become more pronounced, with patients experiencing challenges in attention, executive function, and memory retrieval—issues linked to reduced cholinergic activity. Neuroimaging studies have demonstrated that diminished cholinergic activity correlates with cognitive decline in individuals with PD. The interplay between cholinergic dysfunction and other neurotransmitter systems, such as dopamine, also influences cognitive health. The loss of dopaminergic neurons disrupts the balance of excitatory and inhibitory signals in the brain, further complicating cholinergic signaling pathways [58].

Overall, PD's impact on the cholinergic system leads to significant memory impairments due to neuronal degeneration and reduced ACh levels. This dysfunction disrupts critical molecular pathways necessary for synaptic transmission and plasticity while interacting with alpha‐synuclein pathology and neuroinflammation. Understanding these mechanisms is vital for developing targeted therapeutic strategies aimed at preserving cognitive function in individuals with PD.

Cholinergic dysfunction is closely associated with neuroinflammation in PD, which can adversely affect cholinergic neurons and their ability to release acetylcholine (ACh). This inflammatory environment may inhibit the plasticity of cholinergic circuits, leading to further cognitive impairments. Research indicates that neuroinflammation exacerbates memory deficits by disrupting the normal functioning of cholinergic pathways [94, 102]. Understanding this relationship is crucial for developing effective interventions targeting cognitive decline in PD.

Recent studies have explored cholinergic modulation as a potential therapeutic strategy to alleviate cognitive symptoms in PD. Cholinesterase inhibitors, which enhance ACh availability by preventing its breakdown, have shown promise in improving cognitive function. These treatments aim to bolster cholinergic signaling and restore synaptic plasticity, offering a potential pathway for addressing memory impairments associated with the disease [50]. This approach highlights the importance of targeting cholinergic dysfunction in therapeutic strategies for PD.

### 4.7 Cortical Thinning and Atrophy

Cortical thinning and atrophy are significant structural changes in the brains of individuals with PD, closely linked to cognitive impairments. These changes involve the loss of neuronal density and synaptic connections in various cortical regions, contributing to cognitive decline. Understanding the molecular mechanisms driving these changes is crucial for elucidating their correlation with memory impairment and other cognitive deficits.

Several factors contribute to cortical thinning and atrophy in PD [103]. The progressive degeneration of dopaminergic neurons disrupts neural circuit functioning, leading to alterations in synaptic plasticity and connectivity [104]. Alpha‐synuclein pathology affects not only dopaminergic neurons but also disrupts cortical neuron integrity, leading to cell death and volume loss. Neuroinflammation, driven by activated microglia and astrocytes, creates a pro‐inflammatory environment detrimental to neuronal health, promoting apoptosis and contributing to cortical thinning [8, 81, 102, 105, 106]. Additionally, oxidative stress and excitotoxicity play roles in exacerbating neuronal damage and atrophy [107, 108].

The relationship between cortical thinning and cognitive decline is well established. Studies have shown that reductions in cortical thickness correlate with impairments in executive function, attention, and memory. Regions like the prefrontal cortex and hippocampus, critical for higher‐order cognitive processes and memory, often exhibit significant atrophy in PD. This atrophy disrupts neural circuits responsible for decision‐making and memory consolidation, leading to cognitive deficits [108, 109].

Therapeutic interventions targeting neuroinflammation, oxidative stress, and excitotoxicity may help maintain neuronal health and function. Anti‐inflammatory agents and antioxidants could mitigate the chronic inflammatory state and oxidative stress associated with PD. Lifestyle modifications such as regular physical exercise and cognitive training have demonstrated neuroprotective effects and may help slow the progression of cortical atrophy by promoting neuronal survival and enhancing cognitive reserve [15, 110, 111].

### 4.8 Disrupted Neural Oscillations

Disrupted neural oscillations are a significant factor in the pathophysiology of PD, closely linked to cognitive impairments experienced by patients. Neural oscillations refer to the rhythmic electrical activity in the brain that facilitates communication across various neural networks, essential for cognitive functions and information processing. In PD, changes in these oscillatory patterns can disturb the balance between excitatory and inhibitory signaling, resulting in cognitive deficits such as memory loss. Investigating the molecular mechanisms behind these disruptions is crucial for understanding the complexities of cognitive dysfunction in PD.

The degeneration of dopaminergic neurons in the substantia nigra is a primary cause of disrupted neural oscillations in PD. Dopamine plays a vital role in modulating neural circuit activity, particularly those related to motor control and cognition. In healthy brains, dopamine ensures a balanced interaction between excitatory and inhibitory signals, promoting optimal oscillatory activity. However, the loss of these neurons leads to decreased dopamine levels, disrupting normal oscillatory patterns within critical brain areas such as the cortex, basal ganglia, and thalamus [112].

The interaction between basal ganglia and cortical regions is essential for maintaining coherent neural oscillations. In PD, dysfunction within basal ganglia circuitry alters neuronal firing patterns, leading to abnormal oscillatory activity, especially in the beta frequency range (13–30 Hz) [113]. Increased beta oscillations have been correlated with both motor symptoms and cognitive deficits in PD patients. These irregular oscillations can hinder proper information processing, affecting cognitive functions like attention and decision‐making [112, 114, 115].

Additionally, molecular changes related to ion channels and neurotransmitter receptors contribute to disrupted neural oscillations in PD. The loss of dopamine alters glutamate and gamma‐aminobutyric acid (GABA) receptor activities, which are crucial for balancing excitation and inhibition within neural networks. This dysregulation can result in excessive excitatory signaling or diminished inhibitory control [46]. Furthermore, the accumulation of misfolded alpha‐synuclein disrupts synaptic transmission and plasticity, impairing communication between neural circuits. Neuroinflammation also plays a role by activating glial cells that release pro‐inflammatory cytokines, further disturbing neurotransmitter signaling and contributing to abnormal oscillatory patterns [85].

Oxidative stress plays a significant role in disrupting neural oscillations in PD, primarily through the accumulation of reactive oxygen species (ROS). This cellular stress leads to neuronal damage and impaired function, affecting ion channel and receptor activity, disrupting calcium signaling, and altering gene expression related to synaptic function. The resultant abnormal oscillatory activity further impairs cognitive processes, particularly memory [116]. Increased beta oscillations have been linked to cognitive deficits, especially in tasks requiring attention and working memory. These abnormal patterns hinder the brain's ability to process and integrate information effectively, complicating memory consolidation and retrieval [114, 115].

The impact of disrupted neural oscillations extends beyond cognitive processes, affecting overall neural network dynamics in PD. The interaction between different frequency bands—such as alpha, beta, and gamma oscillations—is crucial for facilitating effective communication among brain regions. Disruptions in these patterns can lead to a breakdown in coordination between neural circuits, further contributing to cognitive decline. Therapeutic strategies aimed at restoring normal neural oscillations are gaining attention, with approaches like deep brain stimulation (DBS) showing promise in modulating abnormal activity within the beta frequency range. DBS has the potential to enhance synchronization of neural activity, which may improve both motor symptoms and cognitive function in PD [117, 118].

Pharmacological interventions targeting the dopaminergic system could also help normalize neural oscillations. Medications that enhance dopamine signaling or modulate glutamatergic and GABAergic transmission may restore the balance between excitation and inhibition, promoting coherent oscillatory activity. Research into compounds specifically targeting the molecular mechanisms underlying disrupted oscillations holds promise for future therapeutic strategies [44, 53, 112, 119].

### 4.9 Gut‐Brain Axis Dysfunction

Gut‐brain axis dysfunction has emerged as a significant area of research in understanding the interplay between the peripheral and central nervous systems, particularly regarding neurodegenerative diseases like PD. The gut‐brain axis refers to the bidirectional communication network linking the gastrointestinal tract and the brain through neural, endocrine, and immune signaling. In PD, disruptions in this axis are associated with both gastrointestinal symptoms and cognitive impairments, suggesting that alterations in gut health may contribute to neurodegenerative processes.

A key factor in gut‐brain axis dysfunction in PD is the role of gut microbiota. The human gut hosts trillions of microorganisms essential for maintaining intestinal health and influencing metabolism, immune function, and neural signaling. Studies have shown significant alterations in gut microbiota composition in individuals with PD, a condition known as dysbiosis. This dysbiosis can lead to an imbalance in microbial metabolites crucial for gut and brain health, such as short‐chain fatty acids (SCFAs), which have neuroprotective effects and influence neurotransmitter systems. Reduced SCFA levels due to dysbiosis may contribute to cognitive decline [66, 120, 121].

The molecular mechanisms underlying gut‐brain axis dysfunction are complex. One critical pathway involves the activation of the vagus nerve, which serves as a primary conduit for communication between the gut and brain. Dysbiosis can lead to pro‐inflammatory cytokine production that activates vagal pathways, resulting in neuroinflammation—a key contributor to neurodegenerative processes in PD. Chronic neuroinflammation can activate microglia and astrocytes, releasing inflammatory mediators that exacerbate neuronal damage and cognitive impairment [122, 123]. Additionally, dysregulation of the hypothalamic‐pituitary‐adrenal (HPA) axis may occur due to gut dysbiosis, leading to increased cortisol levels that have neurotoxic effects [124].

The relationship between gut‐brain axis dysfunction and gastrointestinal symptoms prevalent in PD is also significant. Symptoms such as constipation can exacerbate dysbiosis and impair nutrient absorption, further impacting cognitive function. Moreover, oxidative stress plays a vital role in this dysfunction; increased reactive oxygen species (ROS) production can lead to inflammation and neuronal degeneration within both the gut and brain. This dual impact creates a feedback loop that further impairs communication along the gut‐brain axis and promotes cognitive decline [24, 66, 96].

Understanding gut‐brain axis dysfunction has important therapeutic implications for PD. Interventions aimed at restoring gut health through probiotics, prebiotics, or dietary modifications may mitigate cognitive impairment by enhancing microbial diversity and promoting beneficial bacteria growth. Anti‐inflammatory agents targeting gut inflammation could also support cognitive health by reducing neuroinflammation burdens [125]. Dietary approaches like the Mediterranean or ketogenic diets have shown promise in positively influencing gut microbiota and neuroinflammation, potentially providing new avenues for intervention in PD [126, 127, 128].

### 4.10 Impairment of Synaptic Plasticity

Impairment of synaptic plasticity is a critical factor in the cognitive deficits observed in PD, particularly affecting learning and memory. Synaptic plasticity, which involves the strengthening or weakening of synapses in response to activity levels, is essential for effective information processing. In PD, this mechanism is disrupted due to various molecular changes arising from the degeneration of dopaminergic neurons.

At the molecular level, synaptic plasticity involves a complex interplay of neurotransmitter systems, ion channels, intracellular signaling pathways, and gene expression mechanisms. The loss of dopaminergic signaling disrupts synaptic transmission homeostasis, altering the expression and functioning of key receptors involved in plasticity, such as NMDA and AMPA receptors [47, 101, 116]. Altered intracellular calcium dynamics also play a role, as reduced dopaminergic signaling can lead to dysregulation of calcium influx, inhibiting the activation of calcium‐dependent kinases necessary for long‐term potentiation (LTP) induction [21].

In addition to these changes, inflammatory pathways and oxidative stress contribute to synaptic plasticity impairment in PD. Neuroinflammation creates a toxic environment for neurons, with cytokines like TNF‐α disrupting AMPA receptor trafficking and surface expression, leading to reduced synaptic efficacy and impaired LTP [8, 80, 98]. Oxidative stress, resulting from mitochondrial dysfunction, alters signaling pathways governing synaptic plasticity, disrupting postsynaptic density and impairing signaling [129, 130].

Gene expression alterations, such as epigenetic modifications, also contribute to synaptic plasticity impairment. These modifications can dysregulate immediate early genes essential for LTP, hindering memory processes [131, 132]. Furthermore, neurotransmitter system dysfunction extends beyond dopamine, with serotonergic and glutamatergic dysregulation exacerbating memory deficits [58].

Overall, the impairment of synaptic plasticity in PD arises from a combination of factors including dopaminergic degeneration, altered calcium dynamics, neuroinflammation, oxidative stress, gene expression changes, and neurotransmitter system dysfunction. Understanding these molecular pathways is essential for developing targeted therapeutic strategies aimed at restoring synaptic function and improving cognitive outcomes in PD. Furthermore, the presence of alpha‐synuclein pathology at synaptic terminals has been identified as an early event in PD pathogenesis. This “synaptopathy” can occur before significant neuronal loss, indicating that early interventions targeting synaptic health may be beneficial [3]. The relationship between synaptic plasticity and cognitive function underscores the need for continued exploration into potential therapeutic interventions.

By unraveling the molecular links between PD and synaptic impairment, researchers may identify potential interventions that could mitigate memory deficits and enhance the quality of life for those affected by this debilitating condition. As our understanding of these intricate mechanisms expands, there is hope for innovative strategies that could effectively address the cognitive challenges faced by individuals with PD.

### 4.11 Altered Neurotransmitter Systems

Altered neurotransmitter systems play a crucial role in the cognitive decline observed in PD. The primary neurotransmitter affected is dopamine, but other neurotransmitters such as acetylcholine, serotonin, norepinephrine (NE), and glutamate are equally important.

Dopamine, synthesized in the substantia nigra, is essential for motor control, motivation, and reward. In PD, the progressive loss of dopaminergic neurons leads to a significant reduction in dopamine levels, particularly in the striatum and prefrontal cortex, areas integral to cognitive function. This decrease impairs synaptic plasticity, necessary for memory formation, by dysregulating D1 and D2 receptor pathways, leading to impaired long‐term potentiation (LTP) and facilitating long‐term depression (LTD) [55].

Acetylcholine (ACh) plays a significant role in memory and learning, particularly in the hippocampus and neocortex. In PD, the degeneration of cholinergic neurons in the basal forebrain reduces ACh release, leading to deficits in attention, learning, and memory. ACh exerts its effects through nicotinic and muscarinic receptors, which modulate synaptic plasticity. The impairment of cholinergic signaling disrupts these receptor pathways, reducing synaptic plasticity and contributing to memory deficits [58, 133].

Serotonin, synthesized in the raphe nuclei, modulates mood, cognition, and behavior. In PD, alterations in serotonergic signaling occur, particularly in the striatum and cortex. The loss of serotonin can result in mood disorders and cognitive impairments. Dysregulation of the serotonin transporter (SERT) and receptor expression, such as changes in 5‐HT2A and 5‐HT1A receptors, affects synaptic plasticity and contributes to cognitive deficits [134].

Norepinephrine (NE), synthesized in the locus coeruleus, is critical for cognition and attention. In PD, the loss of noradrenergic neurons leads to cognitive dysfunction, including impaired memory retrieval and executive function. NE acts through α and β adrenergic receptors, which modulate neuronal excitability and synaptic plasticity. The loss of NE disrupts these signaling pathways, impairing synaptic function and memory processes [135, 136].

Glutamate, the primary excitatory neurotransmitter, is significantly impacted in PD. Dysregulation of glutamatergic signaling can lead to excitotoxicity, disrupting the balance between excitatory and inhibitory signaling. This contributes to cognitive deficits by impairing NMDA receptor activation, necessary for LTP induction, and enhancing long‐term depression (LTD) [47].

The interplay among these altered neurotransmitter systems creates a complex network contributing to cognitive deficits in PD. Understanding these molecular mechanisms is crucial for developing therapeutic strategies aimed at restoring neurotransmitter balance and improving cognitive outcomes in individuals with PD.

**Figure 1:**
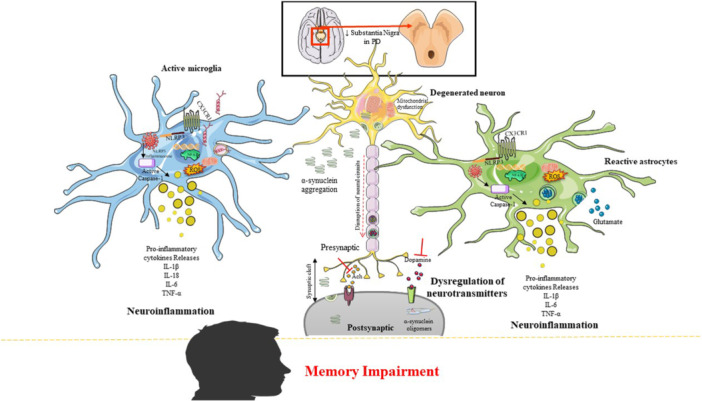
Molecular mechanisms associated with memory impairment in PD.

Figure 1 and Table 1 provides an overview of the molecular mechanisms associated with memory impairment in PD.

**Table 1:** Molecular processes involved in memory impairment associated with PD.

Molecular mechanism	Effect	References
Disruption of neural circuits	– Parkinson's Disease (PD) affects dopaminergic neurons, disrupting neural connectivity and cognitive function. – Loss of dopamine impacts memory formation and retrieval, particularly in the striatum and prefrontal cortex. – α‐synuclein aggregates interfere with neurotransmitter dynamics and contribute to neuroinflammation. – Dysregulation of glutamatergic signaling leads to excitotoxicity, impairing synaptic plasticity. – Cholinergic system loss affects attention and learning. – Neurotrophic factors like BDNF are reduced, impairing synaptic maintenance. – Lifestyle factors such as exercise can enhance neuroplasticity and cognitive outcomes.	[41, 42, 43, 44, 45, 46, 47, 48, 49, 50, 52, 53, 54, 137]
Dopamine depletion	– Dopamine depletion is central to PD, affecting both cognitive and motor functions. – Loss of dopaminergic neurons in the substantia nigra leads to decreased dopamine levels across the brain. – α‐synuclein aggregation disrupts neuronal processes and contributes to mitochondrial dysfunction. – Neuroinflammation exacerbates dopamine loss through microglial activation. – Excitotoxicity from glutamate dysregulation further impairs dopaminergic neurons. – Dopamine is crucial for memory encoding and retrieval; its depletion leads to significant cognitive deficits.	[55, 56, 57, 58, 59, 60, 61, 62, 138]
Oxidative stress	– Oxidative stress results from an imbalance between ROS production and detoxification, contributing to neuronal degeneration in PD. – Mitochondrial dysfunction leads to increased ROS and neuronal damage. – Neurotoxic substances exacerbate oxidative stress by targeting dopaminergic neurons. – Antioxidant defenses are often impaired in PD, reducing the ability to combat oxidative stress. – Oxidative stress affects neurogenesis and synaptic plasticity, further impairing cognitive function.	[65, 66, 67, 68, 69, 70, 71, 72, 73, 74, 75, 76, 77, 92, 120, 139]
Neuroinflammation	– Neuroinflammation is characterized by glial cell activation, leading to a chronic inflammatory state that exacerbates neuronal damage in PD.	[45, 72]
Alpha‐synuclein pathology	Alpha‐synuclein misfolding and aggregation into Lewy bodies is central to Parkinson's Disease (PD), affecting neuronal health and cognitive function. Pathological changes are influenced by genetic mutations and environmental factors, leading to neurotoxicity and mitochondrial dysfunction. The spread of pathology occurs in a prion‐like manner, impacting cognitive functions in the prefrontal cortex and hippocampus. Targeting alpha‐synuclein may improve cognitive outcomes in PD.	[11, 52, 69, 71, 80, 85, 90, 91, 92, 93, 94, 95, 96, 98, 99, 100]
Cholinergic system dysfunction	PD disrupts cholinergic function due to degeneration of cholinergic neurons in the basal forebrain, leading to decreased acetylcholine (ACh) levels. This reduction impairs synaptic plasticity and neurotransmission, exacerbating cognitive decline. Cholinergic dysfunction interacts with alpha‐synuclein pathology and neuroinflammation, further impairing memory. Cholinesterase inhibitors may help improve cognitive function by enhancing ACh availability.	[12, 50, 58, 94, 100, 101, 102]
Cortical thinning and atrophy	Significant structural changes in PD include cortical thinning and atrophy due to neuronal degeneration and neuroinflammation. These changes correlate with cognitive decline, particularly in the prefrontal cortex and hippocampus. Therapeutic strategies targeting neuroinflammation and oxidative stress may help maintain neuronal health and cognitive function. Lifestyle modifications may also slow cortical atrophy progression.	[8, 15, 81, 102, 105, 106, 107, 108, 110, 111]
Disrupted neural oscillations	Disrupted neural oscillations in PD affect cognitive functions due to altered excitatory and inhibitory signaling. Dopaminergic neuron degeneration leads to abnormal oscillatory patterns that hinder information processing. Neuroinflammation and oxidative stress contribute to these disruptions. Therapeutic strategies like deep brain stimulation (DBS) aim to restore normal oscillations and improve both motor symptoms and cognitive function in PD.	[46, 85, 112, 116]
Gut‐brain axis dysfunction	Gut‐brain axis dysfunction involves communication between the gastrointestinal tract and the brain. In PD, disruptions in this axis are linked to gastrointestinal symptoms and cognitive impairments. Understanding this relationship is crucial for exploring potential therapeutic strategies targeting gut health to mitigate neurodegenerative processes associated with PD.	[24, 66, 96, 120, 121, 124, 126, 127, 128]
Impairment of synaptic plasticity	– Impairment of synaptic plasticity is crucial in cognitive deficits seen in PD, particularly affecting learning and memory. – Synaptic plasticity involves the strengthening or weakening of synapses based on activity levels. – Dopaminergic neuron degeneration disrupts neurotransmitter systems, affecting NMDA and AMPA receptors critical for plasticity. – Altered calcium dynamics inhibit long‐term potentiation (LTP) induction. – Neuroinflammation and oxidative stress further impair synaptic plasticity by disrupting AMPA receptor trafficking and signaling pathways. – Epigenetic modifications can dysregulate immediate early genes essential for LTP, hindering memory processes. – Dysfunction in serotonergic and glutamatergic systems exacerbates memory deficits.	[3, 8, 21, 47, 58, 101, 116, 131]
Altered neurotransmitter systems	– Altered neurotransmitter systems significantly contribute to cognitive decline in PD. – Dopamine depletion affects motor control and cognitive functions due to loss of dopaminergic neurons in the substantia nigra. – Acetylcholine is vital for memory; its reduction leads to deficits in attention and learning. – Serotonin alterations can result in mood disorders and cognitive impairments through dysregulation of serotonin transporters and receptors. – Norepinephrine loss leads to cognitive dysfunction affecting memory retrieval and executive function. – Glutamate dysregulation can cause excitotoxicity, impairing NMDA receptor activation necessary for LTP induction.	[55, 58, 133, 134, 135]

## 5 Conclusion

PD is characterized by a complex interplay of molecular mechanisms that contribute to cognitive decline, particularly memory impairment. This condition is not solely a motor disorder but also involves significant cognitive deficits. The disruption of neural circuits, primarily due to dopamine depletion, plays a central role in both motor and cognitive functions. Additionally, neuroinflammation and oxidative stress exacerbate these deficits, highlighting the impact of systemic factors on disease progression.

The involvement of alpha‐synuclein pathology and cholinergic dysfunction adds complexity to the cognitive deficits in PD. Alpha‐synucleinopathy, especially in the hippocampus, can lead to early memory impairment and altered synaptic transmission. Cholinergic disturbances within brainstem and corticostriatal pathways are also implicated in cognitive deficits. Furthermore, structural changes such as cortical thinning and atrophy affect neural oscillations and synaptic plasticity, contributing to cognitive decline.

Understanding these mechanisms underscores the need for a comprehensive approach to treating PD. Future research should focus on elucidating these pathways further and exploring interventions that can mitigate cognitive decline. By addressing both motor and non‐motor symptoms, we can enhance the quality of life for individuals with PD. This includes considering novel insights from the dysfunction of the gut‐brain axis and alterations in neurotransmitter systems, which may provide new avenues for therapeutic strategies.

## Author Contributions

All authors contributed to the conception and design of this narrative review. **Yousef Baghcheghi**, as the corresponding author and supervisor, led the project, oversaw all stages from ideation to final submission, provided critical guidance, and substantially contributed to manuscript revision and editing. **Mohammad Pourfridoni** played a major role in writing the initial draft, extensive editing, and revising the manuscript, particularly in response to reviewer comments. **Zohreh Hakemi** contributed significantly to the literature search, data collection, and preparation of the first draft of several sections. **Habibeh Mashayekhi‐sardoo** assisted in preparing and designing the figures and tables. All authors reviewed, provided feedback on successive drafts of the manuscript, and read and approved the final version.

## Funding

The authors have nothing to report.

## Conflicts of Interest

The authors declare no conflicts of interest.

## Transparency Statement

The lead author Yousef Baghcheghi affirms that this manuscript is an honest, accurate, and transparent account of the study being reported; that no important aspects of the study have been omitted; and that any discrepancies from the study as planned (and, if relevant, registered) have been explained.

## Data Availability

This manuscript constitutes a narrative review of existing scientific literature. Consequently, no new primary data were created or analyzed as part of this study.
